# USP19 regulates DNA methylation damage repair and confers temozolomide resistance through MGMT stabilization

**DOI:** 10.1111/cns.14711

**Published:** 2024-04-21

**Authors:** Jiaqi Liu, Kaikai Wang, Qian Zhu, Yixin Zhang, Yuping Chen, Zhenkun Lou, Jian Yuan

**Affiliations:** ^1^ Department of Plastic and Reconstructive Surgery, Shanghai Ninth People's Hospital Shanghai Jiao Tong University School of Medicine Shanghai China; ^2^ Department of Oncology Mayo Clinic Rochester Minnesota USA; ^3^ Department of Neurosurgery, The Second Affiliated Hospital, School of Medicine Zhejiang University Hangzhou China; ^4^ Department of Radiation Oncology, Ruijin Hospital Shanghai Jiaotong University School of Medicine Shanghai China; ^5^ State Key Laboratory of Cardiology and Research Center for Translational Medicine, Shanghai East Hospital Tongji University School of Medicine Shanghai China

**Keywords:** deubiquitination, glioblastoma, MGMT, temozolomide resistence, usp19

## Abstract

**Objective:**

To elucidate the relationship between USP19 and O(6)‐methylguanine‐DNA methyltransferase (MGMT) after temozolomide treatment in glioblastoma (GBM) patients with chemotherapy resistance.

**Methods:**

Screening the deubiquitinase pannel and identifying the deubiquitinase directly interacts with and deubiquitination MGMT. Deubiquitination assay to confirm USP19 deubiquitinates MGMT. The colony formation and tumor growth study in xenograft assess USP19 affects the GBM sensitive to TMZ was performed by T98G, LN18, U251, and U87 cell lines. Immunohistochemistry staining and survival analysis were performed to explore how USP19 is correlated to MGMT in GBM clinical management.

**Results:**

USP19 removes the ubiquitination of MGMT to facilitate the DNA methylation damage repair. Depletion of USP19 results in the glioblastoma cell sensitivity to temozolomide, which can be rescued by overexpressing MGMT. USP19 is overexpressed in glioblastoma patient samples, which positively correlates with the level of MGMT protein and poor prognosis in these patients.

**Conclusion:**

The regulation of MGMT ubiquitination by USP19 plays a critical role in DNA methylation damage repair and GBM patients’ temozolomide chemotherapy response.

## Graphical abstract

Deubiquitinase USP19 directly interacts with and deubiquitinates MGMT, which repairs the DNA methylation damage response. USP19 plays a critical role in DNA methylation damage repair and temozolomide treatment for GBM patients.

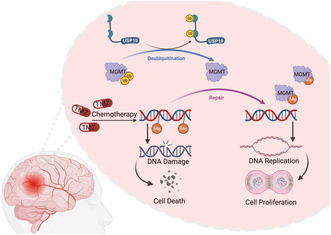

## 1 INTRODUCTION

Glioblastoma (GBM) is a worldwide crucial public health problem, which is the most common and aggressive primary brain malignant tumor.^1^ Although the current standard therapy, including surgery, radiation therapy, and chemotherapy, has been applied to patients, the survival rate of GBM patients is low.^2^, ^3^, ^4^ Temozolomide (TMZ) is an oral alkylating agent and first‐line chemotherapy drug for GBM clinical first‐line chemotherapy drug.^5^ However, almost all patient treatments remain difficult and challenging, who eventually develop resistance after being treated with TMZ for months.^6^, ^7^ Moreover, recurrent GBM patients in general are more difficult to get satisfactory efficacy through the standard treatment.^8^ Therefore, understanding the mechanism of GBM resistance to TMZ is the most important solution for improving TMZ treatment effectiveness.^9^

There are several factors that have been associated with TMZ resistance. However, O6‐methylguanine‐DNA methytransferase (MGMT) removes the alkyl group from the lesion site on DNA, which is the major reason for TMZ resistance.^10^ MGMT is a DNA repair protein, which repairs the naturally occurring mutagenic or TMZ‐induced DNA lesion O6‐methylguanine (O6MG) back to guanine and prevents mismatch and errors during DNA replication and transcription.^10^ As the key regulatory mechanism of DNA methylation repair, the regulation of MGMT levels is an important process in this pathway. The key regulatory mechanism of the levels of MGMT is the ubiquitin‐mediated proteolytic degradation which is an important post‐translational modification for the pathway of direct repair of O6‐alkylation lesions by human MGMT.^11^, ^12^ Ubiquitination is a type of protein post‐translational modification by adding ubiquitin, which is a universal regulatory protein that has been found in all tissues.^13^ In contrast, deubiquitinating enzymes (DUBs) are proteases that process ubiquitin or ubiquitin‐like gene products, reverse the modification of proteins by a single ubiquitin, and remodel polyubiquitin chains on target proteins.^14^ Previous studies showed that the ubiquitin‐conjugating enzyme regulates the ubiquitination of O6‐methylguanine‐DNA methyltransferase.^11^ But the deubiquitination process that regulates MGMT stabilization in cancer is still unknown.

Here, we report that USP19, a deubiquitination enzyme, regulates MGMT ubiquitination level and human GBM cell sensibility to TMZ through the DNA methylation repair pathway. Mechanistically, USP19 binds to MGMT and then deubiquitinates MGMT, causing the removal of ubiquitin chains, which stabilize MGMT. Furthermore, USP19 overexpression is observed in MGMT‐positive GBM cell lines, and these cell lines lacking USP19 are sensitive to TMZ, and these studies uncover a previously unknown mechanism regulating both O6‐methylguanine‐DNA repair pathway and TMZ resistance of GBM cells.

## 2 RESULTS

### 2.1 USP19 stabilizes MGMT

**FIGURE 1:**
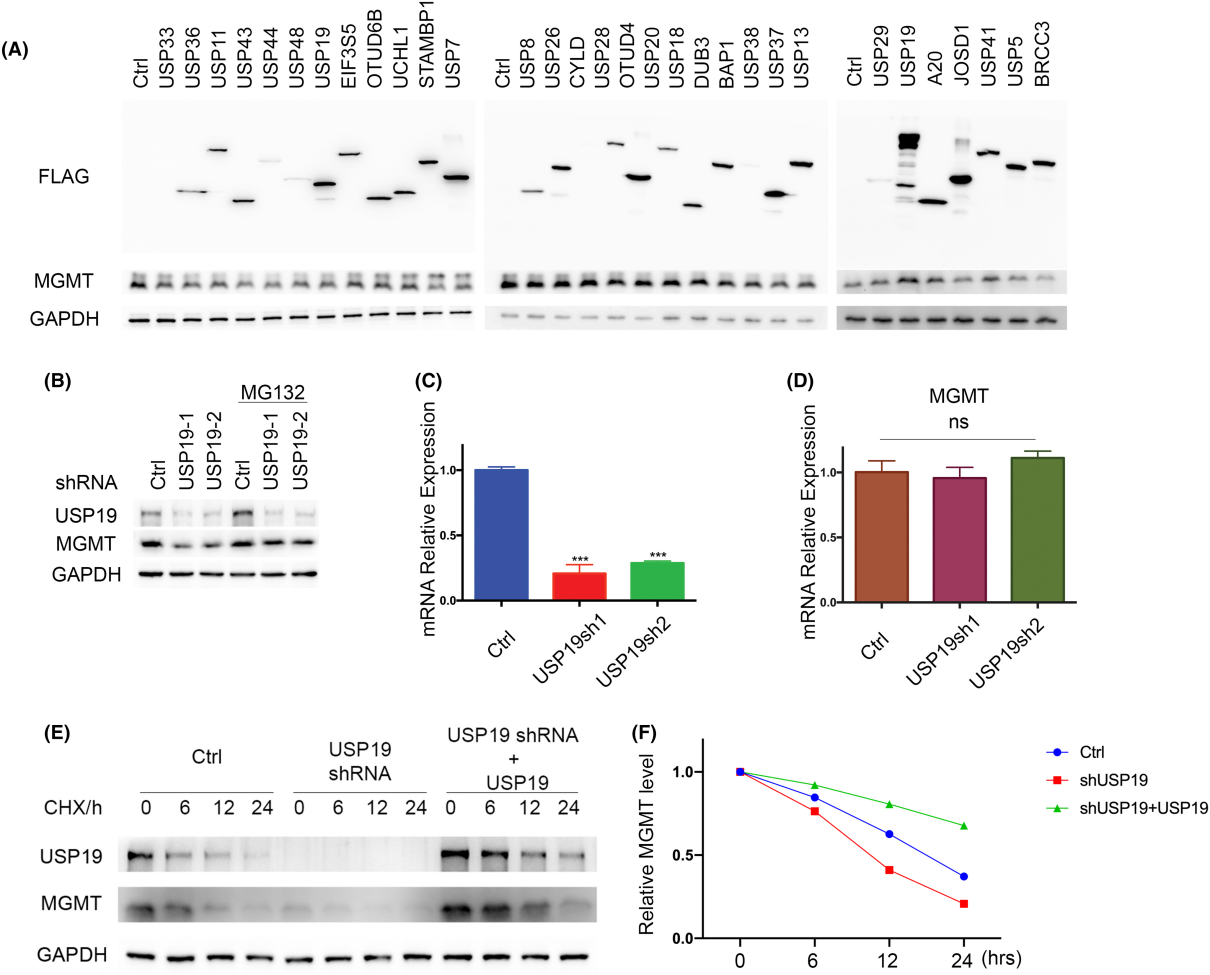
USP19 regulates MGMT protein level. (A) Deubiquitinases (DUBs) were transfected in cells. After 48 h, cells were lysed, and Western blot was performed with the indicated antibodies. (B) Cells stably expressing USP19 shRNAs were treated with vehicle or MG132 for 4 h, and then lysed. Western blot was performed with the indicated antibodies. (C, D) Cells stably expressing control or USP19 shRNAs were harvested for check the mRNAs were extracted from the cells and subjected to qPCR. Error bars represent ± S.E.M. from three independent experiments. ***p < 0.001. Statistical analyses were performed with the Student's *t*‐test. (E, F) Cells stably expressing control shRNA, USP19 shRNA, and USP19 shRNA together with shRNA‐resistant USP19 were treated with cycloheximide (1.0 μg/mL), and harvested at the indicated times. Figure E shows immunoblots of USP19 and MGMT. Figure F shows the quantification of the MGMT level relative to GAPDH.

Following alkylation stress, MGMT functions as a DNA repair enzyme that specifically transfers alkyl adducts from the O6 position of guanine to the cysteine residue in its active site. Their reversible binding of the alkyl group to MGMT protein functionally inhibits its enzyme activity and leads to protein degradation. Previous studies have reported that MGMT is ubiquitinated and degraded through the ubiquitin–proteasome pathway after it repairs the O6‐methylguanine‐DNA lesions. However, how the deubiquitinating process regulates MGMT is unclear. To identify potential DUBs that can deubiquitinate and stabilize MGMT, we overexpressed a panel of deubiquitinases in cells individually and examined the MGMT protein level. As shown in Figure 1A, overexpression of ubiquitin‐specific protease USP19 could dramatically increase MGMT protein level. To further confirm the relationship between USP19 and MGMT, we used two different specific short hairpin RNAs (shRNAs) to deplete endogenous USP19 in T98G cells and then detected MGMT protein level. We found that knockdown of USP19 dramatically decreased MGMT protein level, which can be rescued by proteasome inhibitor MG132 (Figure 1B). As shown in Figure 1C,D, depletion of endogenous USP19 by two USP19‐specific shRNAs markedly decreased protein level, but not the MGMT mRNA level, suggesting that USP19 regulates MGMT through post‐translational modification. To further establish whether USP19 regulates MGMT protein stability, we treated cells with cycloheximide (CHX) and determined the half‐life of MGMT. As shown in Figure 1E,F, MGMT stability was dramatically decreased in USP19‐depleted cells, while reconstitution of USP19 could rescue MGMT protein stability in USP19‐depleted cells. Taken together, these results suggest that USP19 regulates MGMT protein level and stability in a proteasome‐dependent manner and USP19 is a potential DUB for MGMT.

### 2.2 USP19 interacts with MGMT

**FIGURE 2:**
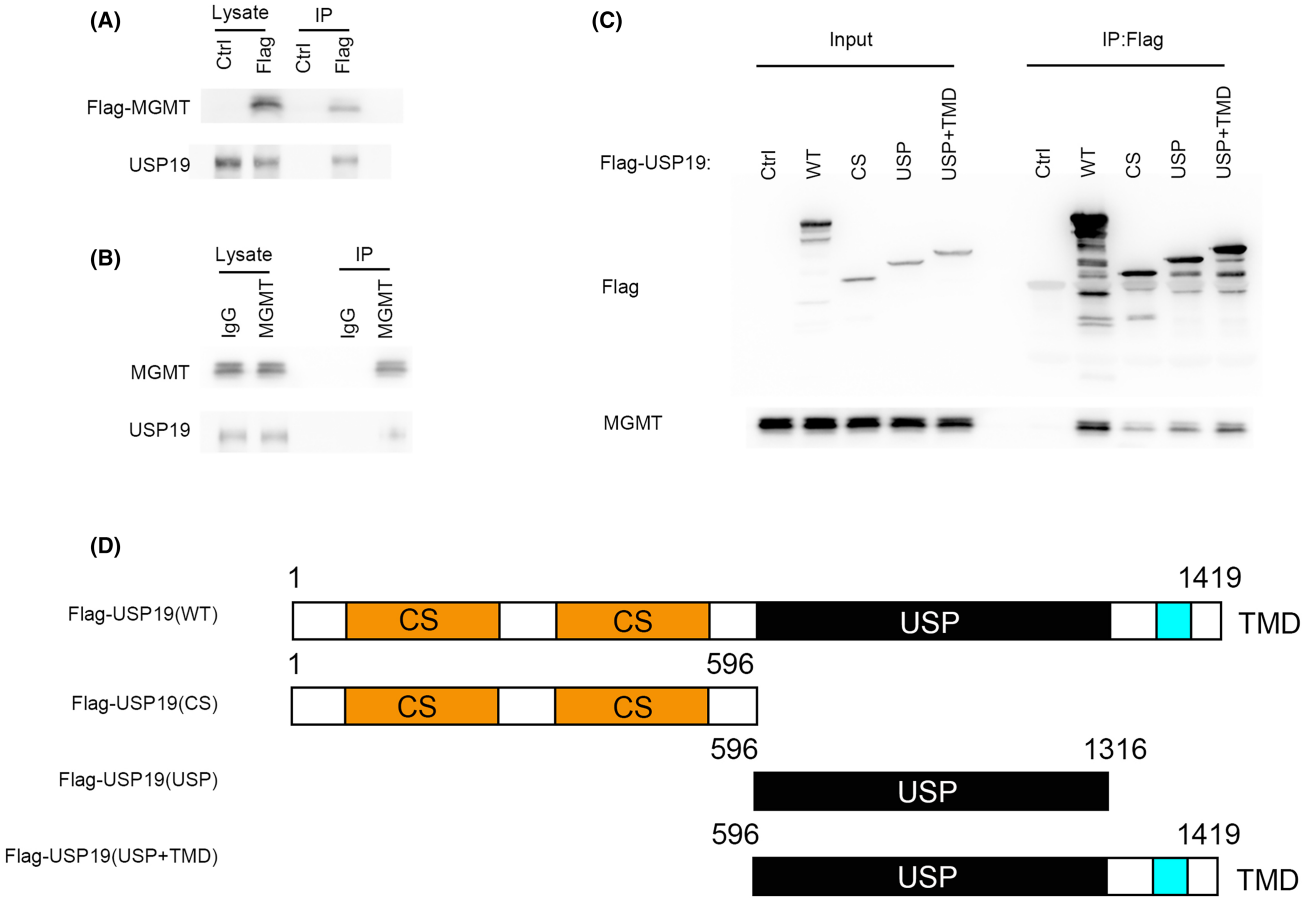
USP19 interacts with MGMT. (A) MGMT interacts with USP19 in cells. Cells were transfected with FLAG‐MGMT. FLAG‐MGMT was purified on anti‐FLAG‐agarose, and then coprecipitating endogenous USP19 was detected by anti‐USP19 antibody. (B) Endogenous MGMT coprecipitates with endogenous USP19. Cell lysates were subjected to immunoprecipitation with control IgG. The immunoprecipitates were then blotted with the indicated antibodies. (C) HCT116 cells transfected with FLAG‐tagged USP19 WT, USP19 1‐596(CS), USP19 USP, USP19 USP+TMD mutant were lysed, and then cell lysates were subjected to immunoprecipitation with anti‐FLAG‐agarose. The immunoprecipitates were then blotted with the indicated antibodies. (D) A schematic illustration of the USP19 constructs used in the study.

To further investigate the mechanism by which USP19 regulates MGMT protein level, we tested whether the USP19 interacts with MGMT. As shown in Figure 2A,B, immunoprecipitating FLAG‐tagged MGMT in 293T cells was able to pull down USP19. Additionally, endogenous immunoprecipitation further confirmed that MGMT binds to USP19 (Figure 2B). To further investigate the mechanism of binding between USP19 and MGMT, we found that the N terminal (1‐596aa) and UI domain of USP19 were responsible for binding with MGMT (Figure 2C).

### 2.3 USP19 deubiquitinates MGMT in vivo and in vitro

**FIGURE 3:**
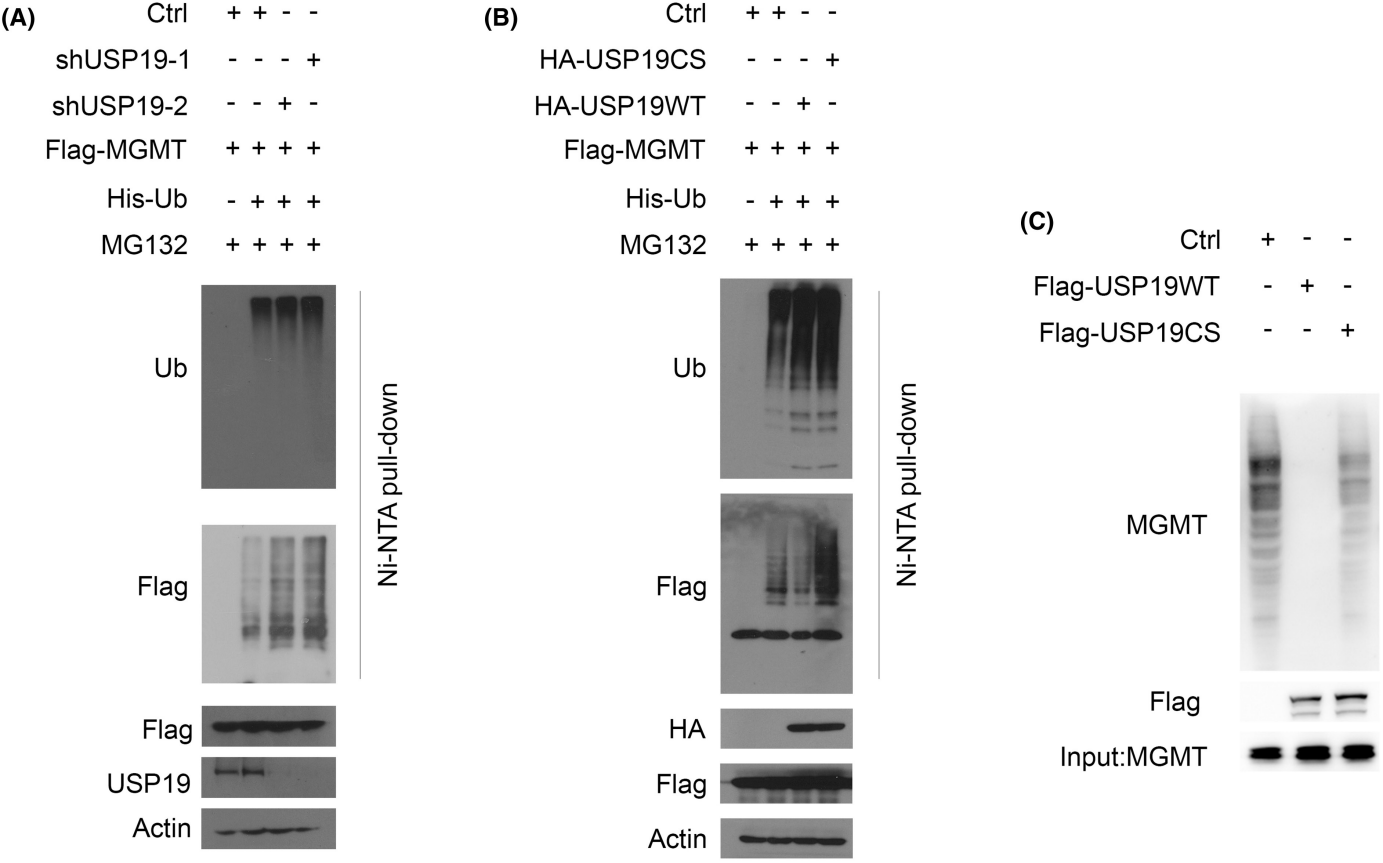
USP19 deubiquitinates MGMT. (A) USP19 deubiquitinates MGMT in cells. Cells stably expressing control or USP19 shRNAs and control or FLAG‐MGMT were transfected with His‐Ub, and then were treated with MG132 for 4 h before harvested. Covalently modified proteins purified on NiNTI‐agarose under denatured conditions. Ubiquitinated MGMT was detected by anti‐FLAG antibody. (B) Cells transfected with indicated constructs were treated with MG132 for 4 h before harvested. Covalently modified proteins were purified on NiNTI‐agarose under denatured conditions and then blotted with indicated antibodies. (C) Deubiquitination of MGMT in vitro by USP19. Ubiquitinated MGMT was incubated with purified USP19WT or USP19CS in vitro and then blotted with indicated antibodies.

To further clarify the effect of USP19‐mediated MGMT stabilization, we assessed whether USP19 deubiquitinated MGMT in cells. As shown in Figure 3A, knockdown of USP19 resulted in a dramatic increase in MGMT polyubiquitination in cell. Conversely, overexpressing USP19 WT in cell resulted in a significant decrease in polyubiquitination of MGMT, while overexpressing USP19 CS mutant failed to alter the level of MGMT polyubiquitination (Figure 3B). To further investigate whether USP19 directly deubiquitinated MGMT, we performed an in vitro deubiquitination assay using 293T cell expressed FLAG‐USP19, S‐MGMT independently and purified by FLAG‐tag, S‐tag affinity agrose beads, respectively. While WT USP19 removed ubiquitin chain from MGMT, the catalytic inactive CS mutant could not (Figure 3C). These results indicate that USP19 is a deubiquitinase for MGMT.

### 2.4 USP19 regulates the sensitivity of MGMT‐positive GBM cell lines to TMZ through MGMT stabilization

**FIGURE 4:**
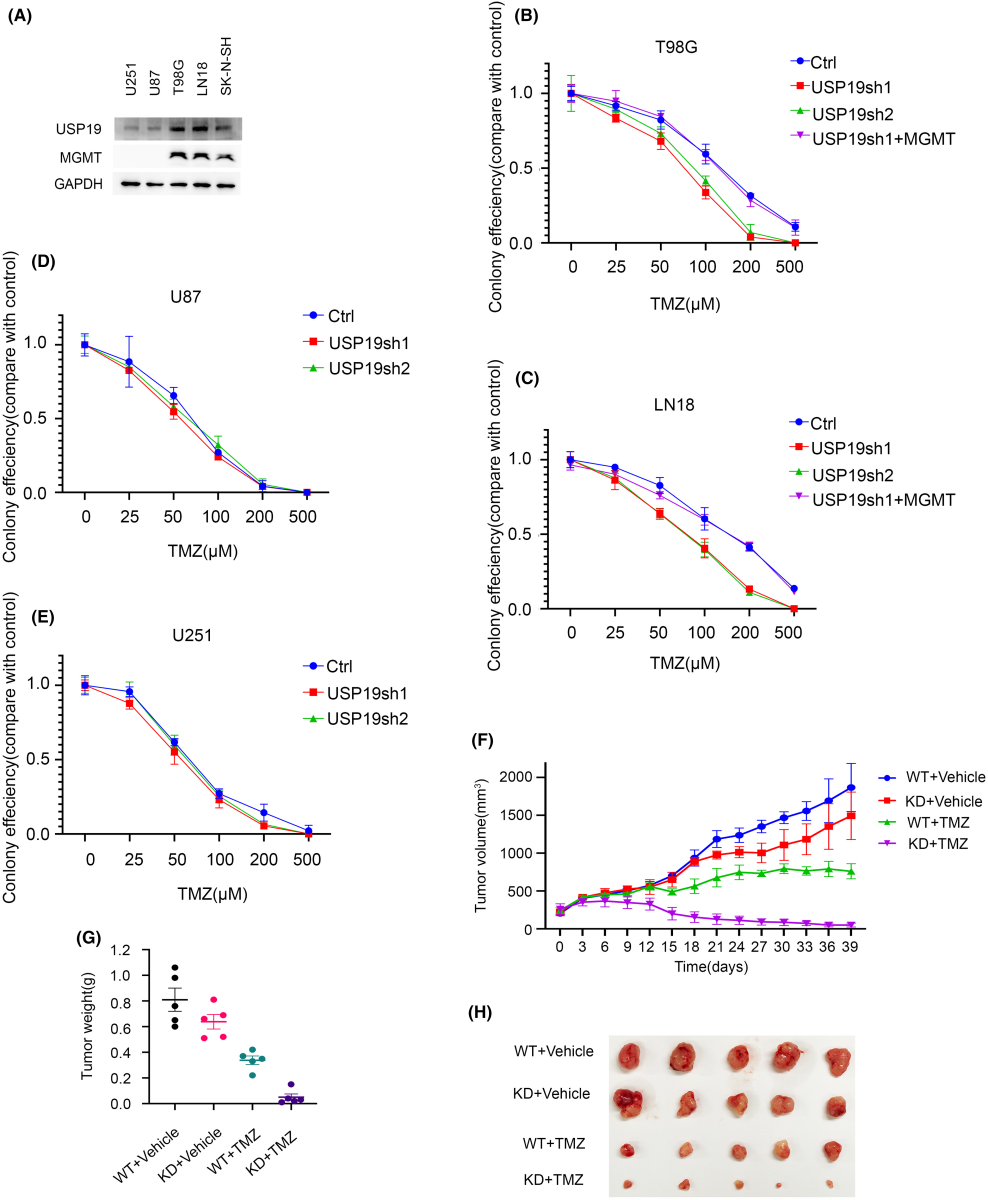
USP19 regulates temozolomide chemotherapy resistance via MGMT. (A) Different brain tumor cells were harvested and lysed, and western blot was performed with the indicated antibodies. (B) T98G, (C) LN18, (D) U87, (E) U251 cells stably expressing indicated constructs were treated with the indicated concentration of temozolomide. Cell survival was performed by colony formation. (F, G) T98G cells stably expressing indicated constructs were subcutaneously injected into the flank of nude mice. Animals were randomized into four groups (*n* = 5) and treated with temozolomide (50 mg/kg) by oral. Tumor volumes were measured by caliper every 3 days, and solid tumor weight was measured at sacrifice. (H) Images of temozolomide‐treated tumor.

It is also known that MGMT plays an important role in resistance of GBM to TMZ. Thus, our hypothesis is that USP19 regulates GBM response to TMZ through MGMT stabilization. To further confirm whether USP19 affects the GBM sensitivity to TMZ, the colony formation assay was performed by T98G, LN18, U251, and U87 cell lines, and these cell lines were separated into two groups depending on the promoter of MGMT gene status, and we checked the MGMT expression status by western blot (Figure 4A). T98G and LN18 are MGMT‐positive cell lines, which had high levels of MGMT activity, and the gene promoter is unmethylated, while U251 and U87 are MGMT‐negative cell lines, which had low levels of MGMT activity and the gene promoter is methylated. All four different cell lines with USP19 knockdown and MGMT‐positive cell lines with MGMT overexpression after USP19 depletion were employed to detect the response to TMZ. As shown in Figure 4B–E, depletion of USP19 sensitized T98G and LN18 cells but not U251 and U87 cells to TMZ. While restoring MGMT expression in USP19 knockdown, T98G and LN18 cells reversed the sensitivity to TMZ (Figure 4B,C). Taken together, these results indicated that USP19 could regulate the sensitivity of MGMT‐positive GBM cell lines to TMZ through MGMT stabilization.

### 2.5 USP19 regulates response of GBM cells to chemotherapy through MGMT stabilization in xenograft

Previous research has shown that MGMT is linked to the therapeutic success of alkylating agent chemotherapy, specifically TMZ treatment. Furthermore, our results show that USP19‐MGMT axis related to the MGMT‐positive GBM cell response to TMZ treatment. To gain more proof to certify the biological function of USP19‐MGMT axis in GBM cells chemo‐resistance in vivo, we employed shRNA to specifically knockdown USP19 in T98G cells, and nude mice were subcutaneously implanted with T98G USP19 depletion cells and control (Ctrl) cells. As shown in Figure 4F–H, depletion of USP19 dramatically sensitized T98G cells to TMZ in flank tumor models. Taken together, our results suggest that USP19 deficiency causes sensitivity of GBM cells to TMZ.

### 2.6 USP19 is positively correlated to MGMT in clinical GBM cancer samples

**FIGURE 5:**
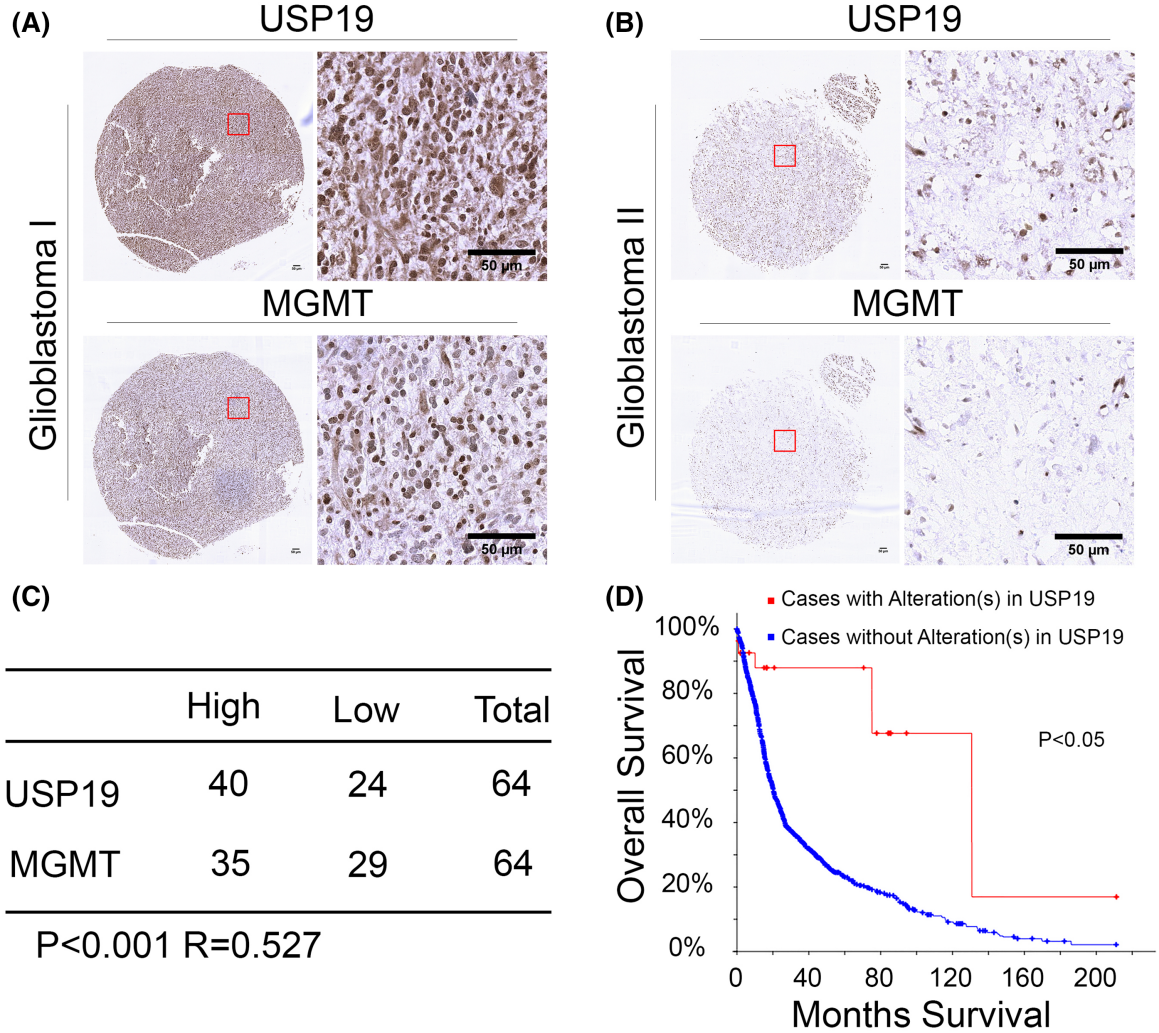
USP19‐deficient glioblastoma sensitizes to temozolomide in vivo. USP19 expression is correlated to MGMT in clinical glioblastoma sample. (A, B) Representative images of immunohistochemistry staining of USP19 and MGMT in glioblastoma. Scar bars, 50 μm. (C) Quantification of USP19 and MGMT protein levels in glioblastoma. Statistical analyses were performed with Chi‐squared test. *R*, Pearson correlation coefficient. (D) Survival analysis of glioblastoma patients was performed. As per data from cbioportal database, patients were categorized into with and without alteration of USP19.

The oral alkylating agent, TMZ, is currently used as first‐line therapy for GBM patients due to its being bioavailable when taken orally, and it is able to cross the blood–brain barrier. After the small molecular TMZ through in the cerebral, it plays the antitumor growth function due to inducing methylation damage in cancer cell DNA. However, MGMT induces drug resistance by removing DNA methylation, which is a major bottleneck for clinical treatment. Previously, some studies showed that expression of MGMT is high level in TMZ resistance cells, and the MGMT gene promoter methylated is the main reason for expression silence. Apart from this, the mechanism of regulating response of unmethylated GBM cells is still unclear. We were interested in investigating whether USP19 relative MGMT expression in clinic GBM samples. As shown in Figure 5A–C, the expressions of USP19 and MGMT in GBM samples were positively correlated. Since USP19‐MGMT axis regulates the sensitivity of GBM to TMZ, we analyzed the overall survival of GBM patients in different USP19 expression backgrounds. As shown in Figure 5D, the data suggest that patients with USP19 alteration have better clinical outcomes. These results suggested that USP19 expression enhanced TMZ stability and induced resistance in GBM, which led to patients having poor treatment outcomes.

## 3 DISCUSSION

Here, we report a clinically actionable approach to regulate the DNA methylation damage repair by MGMT post‐translational modification. Due to the important role of MGMT in DNA methylation damage repair, it has already become a biomarker for distinguishing patients who would benefit from TMZ treatment from those who would not.^10^, ^15^ The specialized regulation of DNA methylation damage repair pathway is the most useful application to improve TMZ treatment efficiency. Previously, some studies uncover that several mechanisms of MGMT inhibitors have been developed sensitizing cells to TMZ.^16^, ^17^ However, we are still unsatisfied with those inhibitors which are less effective, with more side effects and non‐specific targets.

The role of unbiquitination in the MGMT inactivated by O6‐benzylguanine or BCNU has been investigated, and the ubiquitin‐conjugating enzyme E2 B (UBE2B) is a regulator that involves MGMT ubiquitin‐mediated degradation.^11^, ^18^, ^19^ Following further research, the Skp2‐Scf complex is an ub‐conjugation mediator that regulates MGMT protein turnover by phosphorylation of ubiquitin–proteasome pathway. All of these studies demonstrate the important function of ubiquitination and sequential proteolytic degradation in post‐translational modification layer to regulate MGMT activation and inactivation. However, the deubiquitination of MGMT which is the key point of the dynamic degradation process still remains largely unknown.

In this study, we found that the DNA O6‐methylation lesions repair is dependent on the USP19‐MGMT axis, which is a new perspective to intervene in the mechanism of alkylation lesions repair, especially the TMZ treatment for GBM patients. USP19 is a DUB reported to protect certain proteins from degradation through its enzymatic activity when investigating the effect of USP19 on the turnover of specific ERAD substrates and inhibitors of apoptosis (c‐IAPs).^20^, ^21^ USP19 stabilized MGMT and regulated MGMT to mediate DNA methylation damage repair process, further underscoring the dynamic interplay of protein post‐translational modification function on DNA methylation repair. Treatment of USP19‐deficient GBM cells with TMZ reduces the colonic formation capacity and reduces proliferation in a heterotopic xenograft mouse model showing the increased effectiveness of TMZ treatment. All these data elucidated that USP19 plays a critical role in the response of alkylation damage after treatment of GBM with TMZ through MGMT deubiquitination. Other studies have supported the collaboration of TMZ with other target inhibitors, with enrichment for MGMT‐positive GBM. Our study reports the potential of USP19‐MGMT axis as a new chemotherapy target and biomarker in GBM patients. This finding may lead to the development of a USP19 inhibitor to enhance GBM sensitivity to TMZ.

## 4 MATERIALS AND METHODS

### 4.1 Cell culture, constructs, and antibodies

We purchased glioblastoma cell lines T98G, LN18, and neuroblastoma cell line SK‐N‐SH from ATCC. All the cell lines were tested and confirmed by the Mayo Clinic Medical Genome Facility Center. FLAG‐USP19 WT and C506S were purchased from Addgene, and we subcloned it to pCMV‐HA and pLV.3‐FLAG vectors. FLAG‐MGMT WT was purchased from Genescript, and we subcloned it to pLV.3‐FLAG vector. The USP19 constructs were kindly provided by Yihong Ye. U87 and U251 glioblastoma cell lines were kindly provided by Jann N. Sarkaria.

Anti‐USP19 (A301‐586A) antibody was purchased from Bethyl. Anti‐MGMT (E6M7V) antibody was purchased from cell signaling. Anti‐Ub (sc‐8017) antibody was purchased from Santa Cruz. Antibodies against MGMT (MAB16200), HA (H9658), FLAG (F1804), β‐actin (A1978), and β‐Tubulin (T8328) were purchased from Sigma.

### 4.2 RNA interference

USP19 shRNAs were purchased from Sigma: USP19 sh#1, CCGGGCGTGATTTGATTCTGTTGTACTCGAGTACAACAGAATCAAATCACGCTTTTTG; USP19 sh#2, CCGGGCTGATGAACAGCTTTGCATACTCGAGTATGCAAAGCTGTTCATCAGCTTTTTG. We generated the lentiviruses for USP19 shRNAs according to the standard protocol.

### 4.3 Deubiquitination assay in vivo

HEK293T cells transfected with FLAG‐MGMT, and His‐Ub as indicated were incubated with 25 μM MG132. After 4 h incubation, the cells were carefully washed with pre‐chilled PBS for two times and harvested. Cells were lysed in 8 M Urea, 0.1 M NaH_2_PO_4_, 300 mM NaCl, and 0.01 M Tris (pH 8.0). Lysates were briefly sonicated to shear DNA and incubated with Ni‐NTA agarose beads (QIAGEN) for 1–2 h at Room Temperature. Beads were washed five times with 8 M Urea, 0.1 M NaH_2_PO_4_, 300 mM NaCl, and 0.01 M Tris (pH 8.0). Input and beads were boiled in loading buffer and subjected to SDS‐PAGE and immunoblotting.

### 4.4 Deubiquitination assay in vitro

To prepare ubiquitinated MGMT as the substrate and USP19 WT/CS for the in vitro deubiquitination assay, HEK293T cells were transfected with USP19 WT, USP19 CS or His‐Ub and S‐MGMT independently. After 36 h, ubiquitinated MGMT, USP19 WT, and USP19 CS were purified from the cell extracts with anti‐S‐affinity or anti‐FLAG‐affinity column in lysis buffer (50 mM Tris–HCl pH 7.8, 137 mM NaCl, 10 mM NaF, 1 mM EDTA, 1% Triton X‐100, 0.2% Sarcosyl, 1 mM DTT, 10% glycerol, and fresh proteinase inhibitors). Following extensive washing with the FLAG‐lysis buffer, the USP19 WT and USP19 CS proteins were eluted with FLAG‐peptides (Sigma). For the in vitro deubiquitination assay, ubiquitinated S‐MGMT protein was incubated with USP19 in a deubiquitination buffer (50 mM Tris–HCl pH 8.0, 50 mM NaCl, 1 mM EDTA, 10 mM DTT, 5% glycerol) overnight at 16°C.

### 4.5 Cell survival assay

The U87, U251, T98G, and LN18 glioblastoma cell lines are stably expressed with the indicated constructs. For clonogenic assay, 500 cells (T98G and LN18), 3000 cells (U87), and 1000 cells (U251) were seeded in 6‐well plate for 24 h, and then treated with indicated concentration of temozolomide. After 2 weeks, cells were washed with PBS, fixed with methanol, and stained using 0.1% crystal violet, and the colony numbers were counted.

### 4.6 Tumor growth study in xenograft

T98G cells stably expressed the control, and USP19 shRNAs were injected subcutaneously into female athymic nu/nu mice (NCI) flank at 1 × 10^6^ cells/mouse by oral gavage at 50 mg/kg in oral plus, while the control group was treated with the same vehicle. The tumor volumes were measured using digital calipers every 3 days. The experiment protocol was approved by the IACUC at Mayo Clinic.

### 4.7 Tissue microarray

The immunostaining was blindly scored by pathologists. The IHC score is calculated by combining the quantity score (percentage of positive stained cells) with the staining intensity score. The quantity ranges from 0 to 4, that is 0, no immunostaining; 1, 1%–10% of cells are stained; 2, 11%–50% are positive; 3, 51%–80% are positive; and 4, ≥81% of cells are positive. The staining intensity was scored as 0 (negative), 1 (weak), 2 (moderate), and 3 (strong). The score for each tissue was calculated by multiplying the intensity with the quantity score (the range of this calculation was therefore 0–12). An IHC score of 9–12 was considered a strong immunoreactivity; 5–8, moderate; 1–4, weak; and 0, negative. Samples with an IHC score of more than 4 were considered to be high, and less than 4 were considered to be low. The score of tumor tissue was determined by comparing it with the staining intensity of normal tubules on the same slide.

### 4.8 Statistical analysis

Data are presented as the mean ± s.e.m. of three independent experiments for cell proliferation. Data are presented as the mean ± s.d. (*n* = 6) for cell survival assay. Data are presented as the mean ± s.d. of five mice for the tumor growth study in xenograft. The Student's *t*‐test and Chi‐squared test were utilized for the statistical analyses (*p < 0.05; **p < 0.01; ***p < 0.001).

## AUTHOR CONTRIBUTIONS

Z.L. and J.Y. conceived and designed the study. J.L. performed most of the experiments and wrote the manuscript. Y.C. helped with the USP19 WT and CS mutant protein purification. J.Y. and Y.Z. reviewed and edited the manuscript. Q.Z. and P.Y. carried out xenograft experiments. K.W. helped with immunohistochemical staining in specimen microarray. Y.C. provided technical expertise with the protein pull‐down assay. Y.C. and K.L. helped with vector construction.

## CONFLICT OF INTEREST STATEMENT

The authors declare no competing interests.

## Supporting information


Figure S1.

## Data Availability

The data that support the findings of this study are openly available in cBioPortal at https://www.cbioportal.org.
