# Supplementary figures and images for: USP19 regulates DNA methylation damage repair and confers temozolomide resistance through MGMT stabilization
Source: CNS Neurosci Ther. 2024 Apr 21;30(4):e14711. doi: 10.1111/cns.14711 (PMC11033335; doi:10.1111/cns.14711)

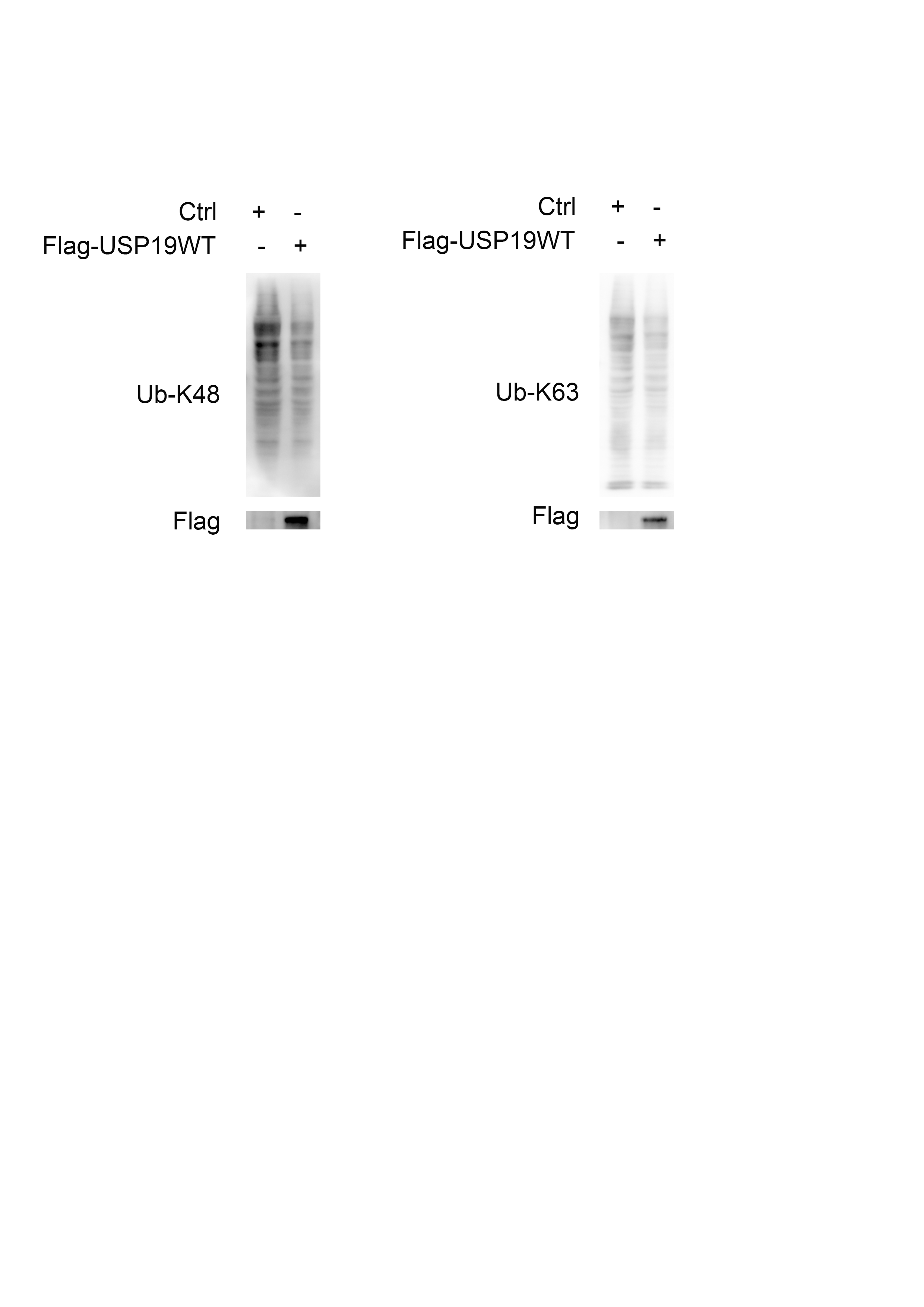

Supplement: Supplementary file 1 — Figure S1. [file CNS-30-e14711-s001.tif]
